# Establishment of spontaneously immortalized Japanese eel muscle-derived preadipocyte cell lines for cultured seafood production

**DOI:** 10.1038/s41538-025-00557-x

**Published:** 2025-11-20

**Authors:** Eriko Kishino, Shizue Saegusa, Daisuke Ikeda

**Affiliations:** 1 Food Technology Research Center, Tokyo Metropolitan Industrial Technology Research Institute (TIRI), Chiyoda-ku, Tokyo Japan; 2 School of Marine Biosciences, Kitasato University, Sagamihara, Kanagawa Japan

**Keywords:** Biochemistry, Cell biology

## Abstract

The lack of immortalized fat cell lines is a significant bottleneck for cultured seafood production. Here, we report three spontaneously immortalized cell lines (JE-KRT224, JE-EK9, and JE-F1140) from the muscle of juvenile Japanese eel (*Anguilla japonica*) capable of robust lipid accumulation. These lines exhibit stable proliferation with good population doubling times and have been cultured for over 120 population doubling levels, indicating spontaneous immortalization. They efficiently absorbed exogenous fatty acid (oleic acid), accumulated intracellular lipids, and developed a fatty acid profile comparable to that of native eel meat. Furthermore, all lines successfully differentiated into adipocytes using standard induction conditions. They expressed the mesenchymal marker vimentin, key mesenchymal stem cell markers (CD29, CD73, CD105), and adipogenic markers (PPARG, FABP4), supporting their characterization as preadipocytes likely derived from mesenchymal stem cells. These novel cell lines represent a valuable resource for advancing fish adipogenesis research and developing multi-component cultured eel meat.

## Introduction

Cultured meat is produced in vitro by cultivating cells harvested from living organisms to generate edible tissue, and is expected to be part of the solution to future food problems because of its environmental advantages, and as it ensures animal welfare and sustainable food supply. In recent years, attempts to enable a wider use of cultured meat have been rapidly progressing, including research into cell culture and suspension culture using microcarriers to enable the mass cultivation of cells, which are the raw material for cultured meat^1,2^, and research regarding edible scaffolds^3–6^, cell sheet engineering^7^, 3D food printing technology^8,9^, and other engineering techniques^10^ to create cultured meat that mimics the biology of living organisms. Although research and development related to cultured seafood has progressed at an astonishing pace, several gaps between research and industrialization have been recognized, such as a limited number of seafood cell lines, limited knowledge of differentiation of cells derived from marine fish, and a lack of serum-free media, demonstrated scaling strategies, and available consumer-ready products^11^.

Meat, which includes terrestrial livestock and seafood, predominantly consists of skeletal muscle, adipose tissue, and connective tissue. Consequently, the development of authentic cultured meat requires suitable cell sources, including myoblasts (for muscle fibers), adipocytes (for fat), and fibroblasts (for connective tissue). However, a significant bottleneck in this field is the limited availability of well-characterized, spontaneously immortalized cell lines, particularly for seafood, which are crucial for scalable and cost-effective production of cultured meat^12,13^. For adipose tissue, a cell line was established from the abdominal subcutaneous adipose tissue of American Yorkshire pigs^14^. In the past 20 years, adipose cell cultures have been reported for eight fish species: Atlantic salmon (*Salmo salar*), European sea bream (*Sparus aurata L*.), and red sea bream (*Pagrus major*). However, all of these were primary cultures obtained from visceral adipose tissue samples^15^. Currently, no fish cell lines are available for adipogenesis research or culturing fish meat. In recent years, a skeletal muscle cell line established from Atlantic mackerel “Mack” cells (*Scomber scombrus*)^12^, a muscle stem cell line from brown-marbled grouper “EfMS” *(Epinephelus fuscoguttatus*)^16^, and a continuous satellite cell line from olive flounder (*Paralichthys olivaceus*)^17^ have been reported. Notably, some fish cell lines, such as Mack and EfMS, have shown the potential for adipogenic differentiation and lipid accumulation when treated with standard adipogenic inducers, including PPARG agonists (e.g., dexamethasone, 3-isobutyl-1-methylxanthine (IBMX), and insulin)^12,16^.

Recently, catches have been declining owing to overfishing and marine pollution^18^. Climate change–related transitions in the marine environment have altered the ecosystems of marine organisms. Furthermore, issues regarding the safety of seafood products, such as the accumulation of microplastics and heavy metal contamination, have also gained attention. Meanwhile, the demand for fish protein is increasing worldwide, and efforts are being made to expand the aquaculture industry to meet this demand. However, it is challenging to sustainably produce fish protein through aquaculture alone^13^ because aquaculture relies on wild fish as raw material and feed. Wild Japanese eels (*Anguilla japonica)* are no exception, and their populations continue to decline, making them endangered species^19^. Japanese eel meat available in the market is made by catching young eels followed by cultivating them for a period and then shipping them^20,21^; therefore, there are concerns regarding the decline in their populations owing to overfishing of glass eels, Moreover, the fluctuation in catch owing to climate change will affect production volume and prices. To achieve a sustainable supply of Japanese eel meat and protect resources while satisfying market demand, efforts are being made to understand the ecology of Japanese eels that migrate from the Mariana Sea to East Asia^22^ and to develop technologies for artificial breeding and full-scale aquaculture, where eels are raised from eggs to adulthood^23^. In addition, if cell culture technology can be applied to produce Japanese eel meat using, we will be closer to achieving sustainability goals and an adequate supply of protein.

Among fish meats, Japanese eel meat has a relatively high fat content. The edible portion of Japanese eels that grow in freshwater areas and are consumed contains approximately 20% fat^23,24^. Adipose tissue and lipid content play a key role in determining the organoleptic properties (flavor, juiciness, texture) and nutritional value of meat in both livestock and fish, and lipid metabolism is closely linked to energy storage, reproduction and survival during long-distance migration, making it important in both basic physiology and applied aquaculture. This underscores the urgent need to establish robust and spontaneously immortalized cell lines from Japanese eels capable of significant lipid accumulation to facilitate research and development of cultured eel meat. Recently, immortalized muscle stem cells from Japanese eels have been established using single-cell cloning^25^. We hypothesized that it would be possible to derive a spontaneously immortalized cell line capable of differentiating into adipocytes from muscle tissue. Accordingly, we attempted to establish a cell line from muscle tissue. This study reports the establishment and comprehensive characterization of three novel, spontaneously immortalized preadipocyte cell lines from Japanese eel muscle. These cell lines offer a unique platform for investigating lipid metabolism in Japanese eels and provide a critical component (adipocytes) for future production of multi-component cultured eel meat.

## Results

### Three spontaneously immortalized cell lines from eel muscle

**Fig. 1:**
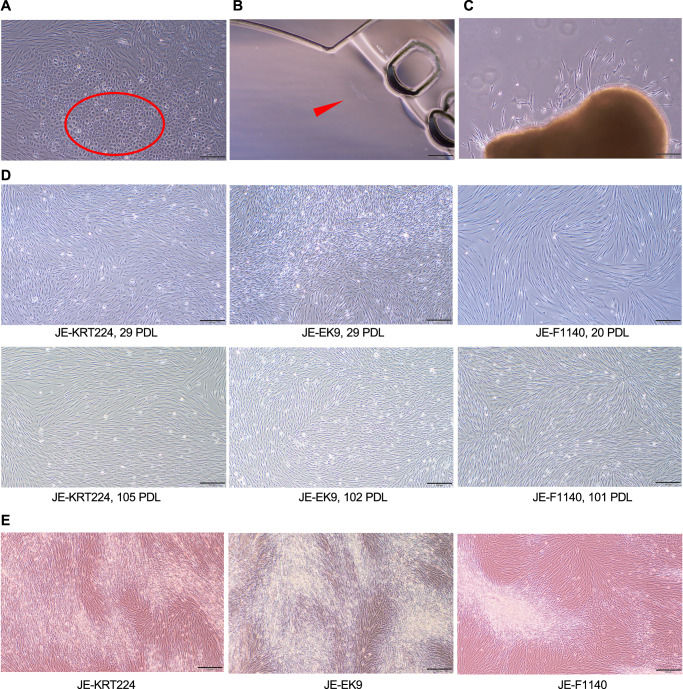
Three spontaneously immortalized cell lines from Japanese eel muscle. **A** Primary culture cells from the Japanese eel muscle (passage 6 after isolation). Several cell types are observed. The circled cell populations were collected by scratching. Scale bar: 200 μm. **B** Cells on sheet-shaped wells. Scale bar: 200 μm. **C** Cells migrating from the primary cell cluster were maintained in culture for 19 months. **D** Cells at approximately 25 and more than 100 divisions, respectively. Scale bar: 200 μm. **E** Overconfluent cells. A multilayered structure was observed. Scale bar: 200 μm.

In this study, we established immortalized cell lines from eel muscle tissue-derived primary cells using two methods (limiting dilution and long-term maintenance culture). Although primary cultures contained various cell types, characteristic cell populations were observed at passage 6 (Fig. 1A). Preliminary experiments (Supplementary Fig. 1) indicated that cells with a relatively round shape and intracellular granules, as highlighted in Fig. 1A (red oval), exhibited lipid accumulation when stained with a fluorescent lipid dye. Morphologically distinct cells were selectively isolated via microscopic scratching for subsequent cloning. JE-KRT224 was obtained using the limiting dilution method in a multicell culture dish (Fig. 1B), and JE-EK9 cells were obtained using the limiting dilution method in 96-well plates. JE-F1140 was established from cells that had migrated out of primary cultured cell clumps that had been maintained for an extended period (19 months) (Fig. 1C). Phase-contrast microscopy images revealed the morphology of the cell lines at approximately 25 and over 100 population doubling levels (PDL) (Fig. 1D). JE-KRT224, JE-EK9, and JE-F1140 cells formed multiple layers after long-term culture (Fig. 1E). To date, JE-KRT224, JE-EK9, and JE-F1140 cells have been cultured over 120 PDL, indicating their spontaneous immortalization. During this study, Mycoplasma contamination of cells was also detected using a MycoAlert™ mycoplasma detection kit (Cat. LT07-218, Lonza, USA) and MycoAlert™ Assay Control Set (Cat. Lonza LT07-518) at approximately 25, 40 PDL and at times exceeding 100 PDL, and negative results were obtained.

### Established cell lines exhibit rapid and stable proliferation

The average population doubling time (PDTs), calculated from the last 10 passages, were 20.8 ± 1.2 (SD) h, 20.2 ± 1.1 h, and 19.3 ± 1.1 h for JE-KRT224, JE-EK9, and JE-F1140, respectively.

**Fig. 2:**
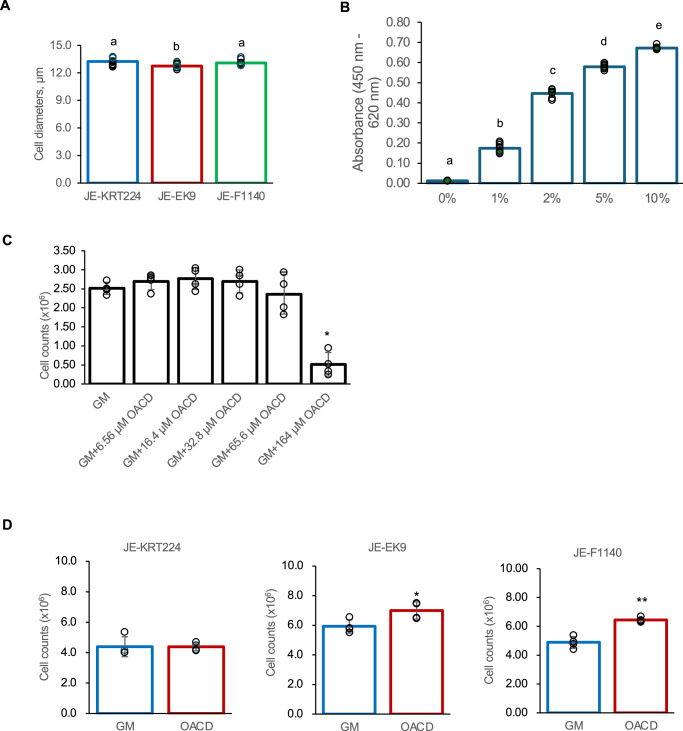
Cell diameter and cell proliferation under various FBS and fatty acid concentrations. **A** The average cell diameter of last 10 passages. Error bars indicate standard deviation, n = 10 distinct samples; statistical significance was calculated by one-way ANOVA with Tukey’s honest significant difference (HSD) post-hoc test (p < 0.05). **B** Cell proliferation when cultured in various concentrations of FBS. Error bars indicate standard deviation, n = 8 distinct samples; statistical significance was calculated by one-way ANOVA with Tukey’s honest significant difference (HSD) post-hoc test (p < 0.05). **C** Cell proliferation in growth medium (GM) or in various concentrations of oleic acid–cyclodextrin complex (OACD) for 7 days. Error bars indicate standard deviation, n = 4 distinct samples; statistical significance was calculated using one-way ANOVA with Dunnett’s test. *p < 0.05. **D** Cell proliferation in GM or GM supplemented with 32.8 μM OACD for 6 weeks. Error bars indicate standard deviation, n = 4; statistical significance was calculated using Student’s t-test. *p < 0.05, **p < 0.01.

The average cell diameters, measured over the last 10 passages, were 13.5 ± 0.4 μm, 12.9 ± 0.3 μm, 13.2 ± 0.4 μm for JE-KRT224, JE-EK9, and JE-F1140, respectively. The average cell diameter of JE-EK9 was smaller than that of E-KRT224 and JE-F1140 (Fig. 2A, p < 0.05).

### Cell lines possess distinct cell surface charge properties

The zeta potential of JE-KRT224, JE-EK9, and JE-F1140 cells was −34.1 ± 3.1 mV, −37.2 ± 2.7 mV, and −15.5 ± 0.4 mV, respectively. The zeta potential of JE-F1140 was significantly different from that of JE-KRT224 and JE-EK9 (*p* < 0.05).

### Cell proliferation depends on serum and is modulated by oleic acid

To determine the optimal concentrations for promoting cell growth, we first assessed the proliferative response of the cell lines to varying concentrations of fetal bovine serum (FBS) and oleic acid. Cell proliferation was evaluated at various FBS concentrations (0, 1, 2, 5, and 10%). JE-KRT224 cells did not proliferate in a serum-free medium (0% FBS), but exhibited growth at FBS concentrations of 1% and above, with maximum proliferation observed at 10% FBS (Fig. 2B).

Cell proliferation was evaluated at various concentrations (6.56, 16.4, 32.8, 65.6, and 164 μM) of oleic acid–cyclodextrin complex (OACD) for 7 days. The addition of OACD at concentrations ranging from 6.56 to 65.6 μM did not significantly affect the proliferation of JE-KRT224 cells over 7 days, whereas a concentration of 164 μM significantly suppressed proliferation (Fig. 2C, p < 0.05).

As the 7-day culture in growth medium supplemented with OACD (6.56–32.8 μM) showed a trend toward better cell proliferation relative to growth medium, the effect of oleic acid on cell proliferation in long-term culture was examined. JE-EK9 and JE-F1140 cells were cultured for 6 weeks in growth medium supplemented with 32.8 μM OACD, and proliferation was found to be significantly higher than that in growth medium without OACD (Fig. 2D, p < 0.05 for JE-EK9 and *p* < 0.01 for JE-F1140). JE-KRT224 cells were partly detached after 6 weeks of culture.

### Cell lines accumulate lipids and display a fatty-acid profile similar to that of native eel meat

**Fig. 3:**
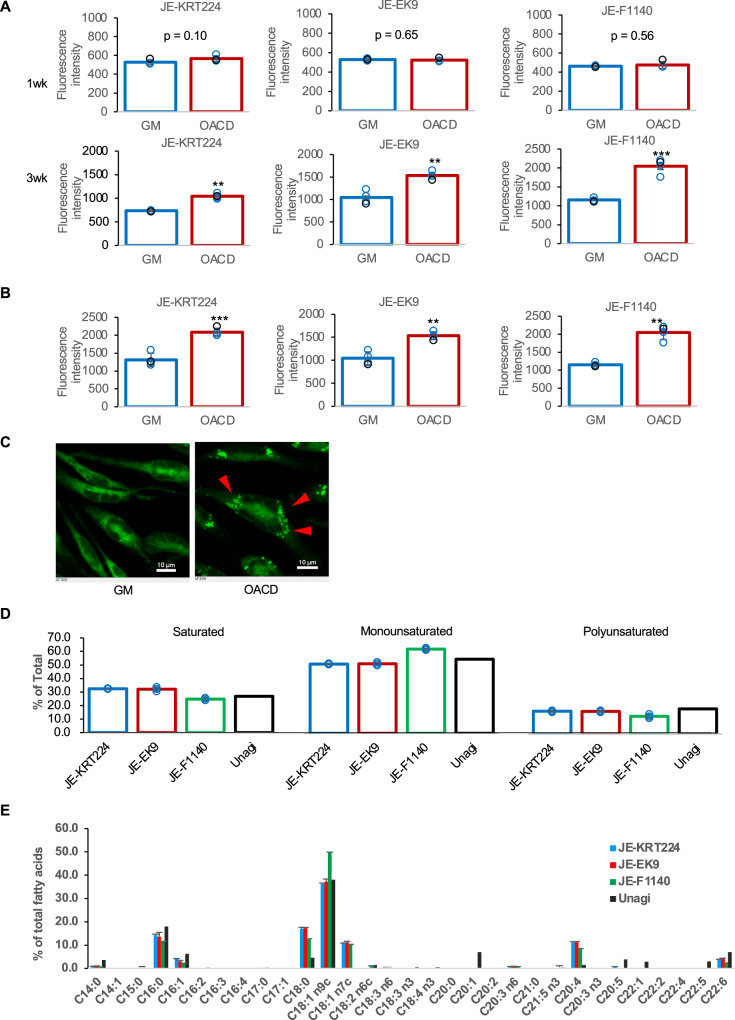
Cell lines accumulate lipids and display a fatty-acid profile similar to that of native eel meat. **A** Lipid accumulation of cells cultured in growth medium (GM) or GM supplemented with 32.8 μM oleic acid–cyclodextrin complex (OACD) at 1 and 3 weeks. Error bars indicate standard deviation, n = 4; statistical significance was calculated using Student’s t-test. **p < 0.01, ***p < 0.001. **B** Fatty acid uptake of cells. Error bars indicate standard deviation, n = 4; statistical significance was calculated using Student’s t-test. **p < 0.01, ***p < 0.001. **C** Cells that have taken up fluorescent probes. Scale bar: 10 μm. **D** Percent of fatty acid saturation in three cell lines and Japanese eel (Unagi) raw meat (adapted from 24), Error bars indicate standard deviation, n = 3. **E** Fatty acid composition of in three cell lines and Japanese eel (Unagi) raw meat (adapted from 24). Error bars indicate standard deviation, n = 3.

Lipid accumulation in cells cultured in growth medium supplemented with 32.8 μM OACD was not significantly different from that in cells cultured in growth medium without OACD after 1 week. However, after 3 weeks, lipid accumulation was significantly higher in cells cultured in medium containing OACD (Fig. 3A).

Cells cultured in growth medium supplemented with 32.8 μM OACD exhibited significantly higher fatty acid uptake than in cells cultured in growth medium (Fig. 3B, p < 0.001 for JE-KRT224, *p* < 0.01 for JE-EK9 and JE-F1140). Representative fluorescence microscopy images confirmed enhanced uptake of a fluorescent fatty acid analog in JE-KRT224 cells cultured in medium containing 32.8 μM OACD (Fig. 3C, arrowheads).

Raw meat of Japanese eel contains approximately 20% fat^23,24^, which significantly impacts flavor and texture. Fatty acid compositions of cells cultured in growth medium supplemented with 32.8 μM oleic acid were analyzed and compared against published data^24^ for cultured Japanese eels. The fatty acid composition of the cells was categorized into saturated fatty acids (SFAs), monounsaturated fatty acids (MUFAs), and polyunsaturated fatty acids (PUFAs) for comparison (Fig. 3D). The fatty acid composition of JE-KRT224 closely resembled that of JE-EK9 cells (Fig. 3E). The proportion of MUFA in JE-F1140 cells was higher than that in JE-KRT224 and JE-EK9 cells (Fig. 3D). Overall, the fatty acid profile of all three cell lines cultured in medium with oleic acid supplementation was similar to that of Japanese eel meat (Fig. 3E).

### Cell lines express mesenchymal- and adipogenic-precursor markers

**Fig. 4:**
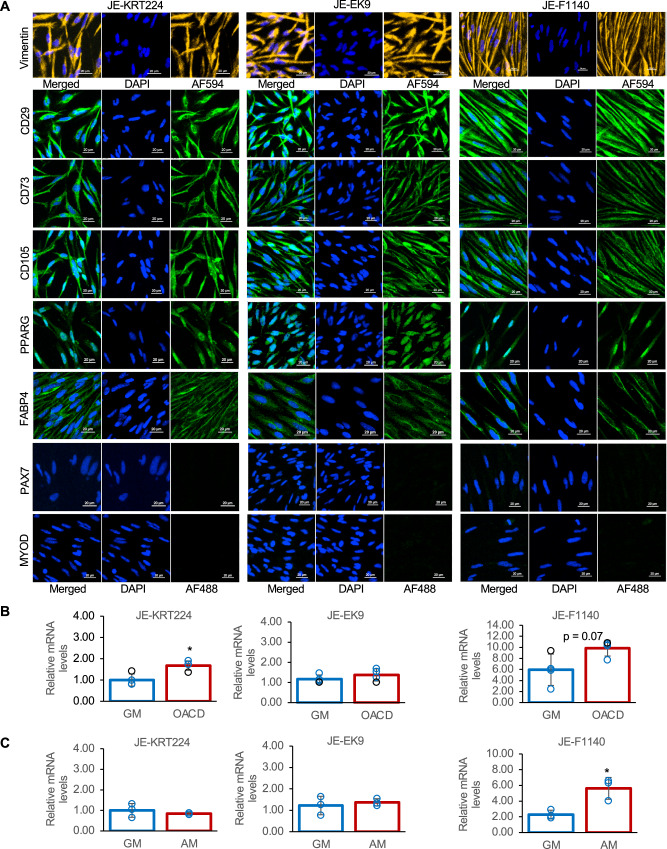
Immunofluorescence staining and changes in PPARG gene expression. **A** Immunofluorescence staining for cell nuclei (DAPI, blue), Vimentin (yellow), CD29, CD73, CD105, PAX7, MYOD, PPARG, and FABP4 (green). Scale bar, 20 μm. **B** RT-qPCR results for the gene expression for PPARG in cells cultured in growth medium (GM) or GM with supplemented with 32.8 μM oleic acid–cyclodextrin complex (OACD). Fold change in expression is represented as 2^−ΔΔCt^. Error bars indicate standard error of the mean, n = 4; statistical significance was calculated using the Student’s t-test. *p < 0.05. **C** RT-qPCR results for the gene expression of PPARG in cells cultured in GM or adipogenic medium (AM; GM with 5 μg/mL insulin, 0.5 mM IBMX, 2.5 μM dexamethasone, and 32.8 μM OACD). Fold change in expression is represented as 2^−ΔΔCt^. Error bars indicate standard error of the mean, n = 3; statistical significance was calculated using the Student’s t-test *p < 0.05.

Immunofluorescence analysis revealed high expression of the mesenchymal stem cell markers CD29, CD73, and CD105, as well as the mesenchymal marker vimentin in all three cell lines (Fig. 4A), indicating that all three cell lines are mesenchymal stem cells (MSCs). Furthermore, the myogenic markers PAX7 and MYOD were not expressed, whereas the adipogenic markers PPARG and FABP4 were expressed even when cultured in growth medium (Fig. 4A).

### PPARG expression is differentially regulated by oleic acid and adipogenic stimuli

PPARG is a key transcription factor that regulates triglyceride and fatty acid metabolism. Given the observed increase in fatty acid uptake, we next examined the expression of PPARG in cells cultured with or without 32.8 μM OACD. *PPARG* mRNA expression in JE-KRT224 cells was significantly upregulated when cultured with 32.8 μM OACD compared with culture in control growth medium (Fig. 4B). In contrast, PPARG expression in JE-EK9 cells was not significantly altered by oleic acid supplementation, whereas JE-F1140 cells showed a trend towards increased PPARG expression when cultured in medium with oleic acid supplementation (p = 0.07) (Fig. 4B). We then investigated PPARG expression under adipogenic differentiation conditions^12^ (growth medium supplemented with insulin, IBMX, dexamethasone, and OACD; hereafter referred to as “adipogenic medium”) relative to standard growth medium. PPARG expression was not significantly altered in JE-KRT224 and JE-EK9 cells cultured in adipogenic medium compared with cells cultured in growth medium. However, JE-F1140 cells exhibited a significant upregulation of PPARG expression in adipogenic medium (Fig. 4C, p < 0.05). These findings suggest differential responsiveness of cell lines to oleic acid supplementation and adipogenic stimuli in terms of *PPARG* gene expression.

### Cell lines selectively differentiate into adipocytes

**Fig. 5:**
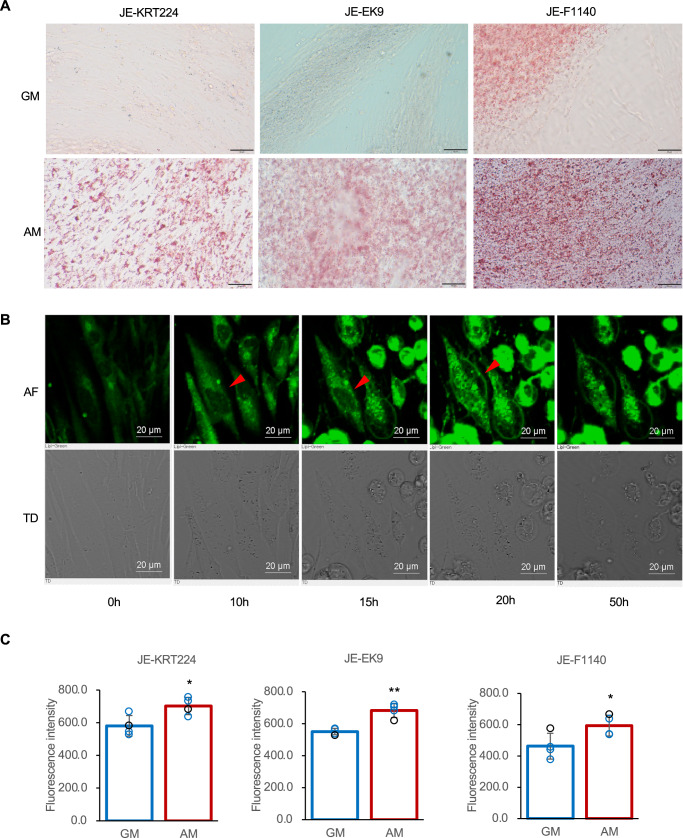
Differentiation potential to adipocytes and lipid accumulation. **A** Oil Red O staining. GM growth medium, AM adipogenic medium (GM with 5 μg/mL insulin, 0.5 mM 3-isobutyl-1-methylxanthine, 2.5 μM dexamethasone, and 32.8 μM oleic acid–cyclodextrin complex). Scale bars, 200 μm. **B** Time-lapse photography of JE-KRT224 cells cultured in AM. upper; AF (autofluorescence; Excitation, 488 nm; Emission, 500–550 nm), lower; TD. Scale bars 20 μm. **C** Lipid accumulation in differentiated adipocytes cultured in GM or AM. Error bars indicate standard deviation, n = 4; statistical significance was calculated using the Student’s t-test *p < 0.05, **p < 0.01.

To assess their multipotency, a characteristic of MSCs^26^, we induced differentiation of the three cell lines into adipogenic, myogenic, chondrogenic, and osteogenic lineages. Under the conditions tested, none of the cell lines differentiated into myotubes, chondrocytes (assessed using Alcian Blue staining), or osteoblasts (assessed using Alizarin Red S staining; Supplementary Fig. 2). However, all three cell lines successfully differentiated into adipocytes, as evidenced by the accumulation of lipid droplets (Fig. 5A). Time-lapse imaging of JE-KRT224 cells cultured in adipogenic medium revealed characteristic morphological changes associated with adipogenesis, including cytoskeletal reorganization and a transition from a fibroblastic to a more rounded shape, with the accumulation of lipid droplets over 60 h (Fig. 5B and Supplementary Movie 1). Quantitative analysis confirmed a significant increase in lipid accumulation in all three cell lines cultured in adipogenic medium for 7 days compared to that in cells cultured in growth medium (Fig. 5C, p < 0.05 for JE-KRT224 and JE-F1140, *p* < 0.01 for JE-EK9).

## Discussion

Cultured meat is expected to contribute significantly to protein supply, environmental conservation, and preservation of endangered species. The availability of suitable cell sources is critical for the production of cultured meat, and spontaneously immortalized cell lines are considered ideal for establishing sustainable systems. However, there is a lack of immortalized cell lines that can be used as raw materials for the production of animal and fish meat^11–13^. To the best of our knowledge, there are no published reports on naturally immortalized fat cell lines for fish. Our study established three novel cell lines capable of accumulating fat from the muscle tissues of juvenile Japanese eels. This is a valuable development in research on cell-cultured fish meat and fat accumulation in fish cells.

When considering industrial-scale production, cells intended as raw materials for cultured meat must be sufficiently tough to withstand subculturing and long-term cultivation. The cells used for the production of cultured meat are primary cells obtained from living tissues, mainly muscles. Primary cells obtained from muscle include multiple cell types, such as myoblasts and fibroblasts; however, weaker cells were selected against with each subculture. With repeated subcultures, fibroblasts, which are the fastest-growing cells, become the dominant cell type^25^. Therefore, we closely observed the cells that survived at the sixth passage of primary cells obtained from the muscle tissue of juvenile Japanese eels and started cloning by targeting the population of cells other than fibroblast-like cells. This strategy resulted in the establishment of the JE-KRT224 and JE-EK9 cell lines. Unlike most mammals, fish grow continuously without any signs of aging^27,28^. Moreover, fish cell cultures tend to grow indefinitely without becoming cancerous, suggesting that fish cells can achieve immortality without becoming malignant^29,30^. JE-F1140 cells were obtained from a cell clump maintained for 19 months by only changing the medium. The ability of the primary cultured cells to sustain for 19 months with only medium changes was noteworthy, as was the observation cell migration from these long-term cultured cell masses, which led to the establishment of the JE-F1140 cell line. The established JE-F1140 cell line had existed for 19 months at the time of cloning, but exceeded 100 PDL even after cloning. Furthermore, the three cell lines showed stable PDT even after exceeding 100 PDL, indicating that spontaneously immortalized cells were obtained.

The culinary appeal of Japanese eel stems from its unique flavor and texture, which are significantly influenced by its high intramuscular fat content. Japanese eels are fatty fish with fat mixed into the muscles, and about 20% of the edible parts of Japanese eels that live in freshwater areas and are used for food is fat^23,24^. Adipose tissue and lipid content play crucial roles in determining the sensory attributes (flavor, juiciness, and texture) and nutritional value of meat, both in livestock and fish. The three cell lines established in this study demonstrated the ability not only to proliferate in the presence of exogenous oleic acid but also to accumulate intracellular lipids concurrently (Figs. 2C and 3A), and the resulting fatty acid profiles were comparable to that of conventionally cultured Japanese eels. This property can be exploited for continuous fat production using established cell lines. Furthermore, the capacity of these cell lines to differentiate into adipocytes when treated with a standard hormonal cocktail (insulin, IBMX, and dexamethasone) indicated their potential to form lipid-laden cells suitable for engineered cultured eel meat constructs.

Animal serum has a complex composition and is expensive, relatively unsafe, and subject to batch variability^31–33^. Therefore, developing a low-cost culture medium consisting of an alternative to FBS and edible components is one of the challenges to make cultured meat feasible. In recent years, efforts have been made to develop a serum-free medium for myoblasts that can be used in serum-free cell-cultured meat^32,33^. Although there are several commercially available serum-free media^34^, currently, there is limited availability of serum-free media suitable for the growth of fish muscle satellite cells or myoblasts, and there have been no studies reporting media suitable for the hybrid culture of myoblasts and preadipocytes^35^. Therefore, FBS was used for cell culture. In this study, 10% FBS was found to be optimal for cell proliferation of JE-KRT224 cells (Fig. 2B). Although JE-KRT224 cells did not proliferate in serum-free medium, their proliferation rate with 2% FBS reached 77% and 86% with 5% FBS reached, relative to the proliferation rate observed with 10% FBS (considered 100%). In addition, it was revealed that oleic acid can promote fat storage and potentially improve cell growth in established cell lines, particularly in long-term cultures (Figs. 2D and 3A). However, it is also important to note that excessive concentrations of oleic acid (e.g., 164 μM) were found to inhibit cell proliferation (Fig. 2C). This suggests an optimal range for oleic acid supplementation that balances lipid accumulation and growth-promoting benefits against potential cytotoxic effects at higher concentrations. It has been shown that high concentrations of fatty acids, including oleic acid, are cytotoxic, and that their effects vary depending on the type and concentration of fatty acids. In particular, oleic acid has a higher fat-forming ability than palmitic acid but causes lesser damage to cells^36–38^. In a study using primary cultured rat hepatocytes, it was found that oleic acid induced cytotoxicity by causing damage to cell membranes^39^. Furthermore, experiments using in human hepatic stellate cells have suggested that oleic acid may cause changes in cytoskeletal proteins, leading to a collapse of cell polarity and ultimately cell death^40^. These findings clearly indicate that the type, concentration, and mixing ratio of fatty acids added to the medium will affect cell proliferation and fat accumulation. As the evaluation of the effect of oleic acid supplementation on established cell lines in this study was limited, a detailed investigation is required for the development of media with compositions suitable for cell proliferation and fat accumulation in established cell lines.

In this study, we could not thoroughly examine the additives in low-serum or serum-free media; therefore, further investigation is required. The established cell lines, characterized by their responses to FBS and oleic acid, could serve as valuable models for the development of serum-free or low-serum media for the culture of fish adipocytes. In this study, insulin, IBMX, and DEX were used to induce adipocyte differentiation. These additives are commonly used to induce fat differentiation in mammalian cells^14,15^ and fish^12,15,16^; however, they cannot be used to produce cultured meat. Therefore, it is necessary to consider the conditions required to induce fat cell formation in edible materials. Genistein, a soy isoflavone that is a type of flavonoid, has been reported to inhibit differentiation of adipose precursor cells (3T3-L1) and reduce the accumulation of lipid droplets^41^. However, evaluation using primary preadipocytes from rainbow trout suggests that 100 μM genistein may promote adipogenesis^11,42^. Therefore, genistein may be an edible component that affects fat accumulation in established cell lines. Furthermore, it is necessary to consider co-culturing myoblasts and preadipocytes, which have different culture conditions in vitro, and to conduct studies regarding the culture of tissues that comprising both muscle and lipid droplets.

Characterization of the three established cell lines suggested that they were committed preadipocytes rather than multipotent MSCs. The cells expressed the mesenchymal cell marker vimentin and MSC markers CD29, CD73, and CD105 (Fig. 4A), indicating their mesenchymal origin. However, their lineage appeared committed towards adipogenesis, which is supported by their successful differentiation into adipocytes under conditions typically used for mammalian cells (Fig. 5A), whereas they failed to differentiate into myotubes, chondrocytes, or osteoblasts under the tested conditions (Supplementary Fig. 2). Furthermore, the lack of expression of key myogenic transcription factors, such as PAX7 and MYOD (Fig. 4A), reinforced their identity as non-myogenic adipogenic precursor cells. Moreover, the observation of dynamic cytoskeletal changes during adipocyte differentiation (Fig. 5B and Supplementary Movie 1) provided further evidence of this process. Although our study confirmed the potential of the three cell lines for lipid accumulation, a more precise quantification of the adipogenic differentiation efficiency, for instance, using flow cytometry, was beyond the scope of this foundational study and remains an important next step for future applications.

Furthermore, the three cell lines took up fatty acids in growth medium containing fatty acids (Fig. 3B), allowing them to accumulate fat, and all three cell lines formed a multilayer structure under in vitro planar culture conditions (Fig. 1E). Their ability to form multilayers in vitro suggests a reduced need for contact inhibition, which could be advantageous for creating dense 3D tissue structures, potentially reducing reliance on complex scaffolding. Providing an appropriate scaffold for cell adhesion may enable a more efficient 3D culture. Cell adhesion to scaffolds is important for adherent cells such as myoblasts and preadipocytes, and cell adhesion to scaffolds followed by proliferation and differentiation are affected by various factors^43,44^, such as cell surface potential^45^ and surface roughness^46^. To investigate the differences in the characteristics of the three cell lines obtained from the muscle tissue of the Japanese eel, we measured their cell surface zeta potential. We found that the zeta potential of JE-F1140 was different from that of JE-KRT224 and JE-EK9. Although the importance of this difference in the cell surface zeta potential is not clear, we hope that this will help understand cell adhesion to plant-based edible scaffolds, which are being considered for use in the production of cultivated meat in the future. In addition, JE-F1140 cells showed a more linear, fibroblast-like appearance than the other two cell lines (Fig. 1D), and the average PDTs calculated from the last 10 passages trend towards the lower side when compared under the same conditions.

In addition, the PPARG response to stimuli differed among the lines, with JE-F1140 showing particularly notable characteristics (Fig. 4B, C). For instance, in JE-KRT224 and, to some extent, JE-F1140 cells, direct oleic acid supplementation of the growth medium significantly upregulated PPARG expression. Interestingly, for JE-KRT224 cells, this effect was more pronounced than the PPARG upregulation observed under standard adipogenic differentiation conditions (which also included oleic acid). JE-F1140, on the other hand, exhibited a strong PPARG upregulation in response to both oleic acid supplementation alone and the complete adipogenic cocktail, highlighting its high responsiveness to adipogenic cues, particularly those involving fatty acids. These distinct PPARG expression patterns further underscore the unique nature of each cell line and suggest potentially different regulatory mechanisms for adipogenesis or lipid metabolism among them. The white adipose tissue of Atlantic salmon contains many fibroblast-like preadipocytes that can differentiate into mature adipocytes in vitro^47,48^. The distinct fibroblastic morphology and rapid proliferation of JE-F1140 cells, along with their unique PPARG response, may indicate that this cell line represents a subpopulation of preadipocytes with characteristics analogous to those of the fibroblast-like preadipocytes reported in the adipose tissue of Atlantic salmon^47,48^. The cell diameter of JE-EK9 was smaller than that of the other two cell lines (Fig. 2A). Overall, it was demonstrated that the three cell lines were distinct cell lines derived from the muscle tissue of the same Japanese eel.

The primary sources of food for Japanese eels are plankton, crustaceans, and fishes found in the sea and rivers^21,22^. These foods contain minimal carbohydrates and sugar, and consist mainly of nutrients other than carbohydrates, such as protein or fat. In addition, Japanese eels do not always have sufficient food in their long-term habitat. Furthermore, to migrate in a state of fasting from East Asia to the Mariana Sea for spawning, they need to store nutrients, especially fat, in their bodies^22^. As mentioned earlier, fat accumulation is an essential survival feature for the successful migration of Japanese eels. The ability of the established cell lines to accumulate lipids when fatty acids are added is thought to be a cellular characteristic of Japanese eels that efficiently utilize the fatty acids derived from the diet.

With Japanese eel myoblast cell lines already established^25^, the preadipocyte cell lines developed in this study provide other key cellular components necessary for producing authentic cultured Japanese eel meat that includes both muscle and fat. These immortalized cell lines are expected to provide insights into the unique fat metabolism of Japanese eels and contribute to aquaculture research. Although these cell lines hold great promise, a more comprehensive evaluation is necessary before considering them for industrial applications. Future investigations should include detailed genomic and chromosomal analyses, such as karyotyping and transcriptomic profiling, to confirm long-term genetic stability and rule out tumorigenic characteristics. Such analyses, although beyond the scope of this initial report, are crucial to ensure the biosafety and consistency necessary for regulatory approval and consumer acceptance. Therefore, this foundational work serves as a crucial first step, providing essential cell materials and basic characterization upon which more rigorous safety and stability assessments can be built in the future.

## Methods

### Primary cell culture, subculture, and maintenance

Glass eel specimens of the Japanese eel (*Anguilla japonica*) were obtained from commercial suppliers, DAIGO TSUSHO Co., Ltd. (Shizuoka, Japan), maintained at 28 °C, and hand-fed daily with regular commercial feed. All animal experiments complied with the regulations of the Animal Experimentation Committee of the School of Marine Biosciences, Kitasato University. Primary cells were isolated using tissue explant techniques, as described previously^25^. The obtained primary cells were maintained in L-15 medium (FUJIFILM Wako Pure Corporation Chemical, Osaka, Japan) supplemented with 15% fetal bovine serum (FBS, Sigma Aldrich, MA, USA), 1% antibiotic–antimycotic mixed stock solution (Cat. 35554-64; Nacalai Tesque, Kyoto, Japan), 1% MEM non-essential amino acid solution (Cat. 139-15651, FUJIFILM Wako Pure Corporation Chemical), and 20 ng/mL bFGF (Cat. KHFGF001; KAC, Kyoto, Japan) at 28 °C without CO_2_. The cells were subcultured once they reached approximately 80%–90% confluence. Cells were maintained under these conditions up to passage three.

From passage four onwards, cells were cultured in a growth medium comprising L-15 medium supplemented with 10% FBS and 1% antibiotic–antimycotic mixed stock solution at 25 °C without CO_2_. When cells reached 80%–90% confluence, they were dissociated using 0.25% trypsin-EDTA solution (Nacalai Tesque) and then subcultured. After trypsinization and cell counting, 5.0–7.0 × 10^5^ cells were seeded into new T75 flasks. Cells were also cryopreserved in CELLBANKER ® 1 plus (Cat. 11912; Zenoaq, Fukushima, Japan) for long-term storage.

### Cell cloning

Cloning was performed using cells at passage 6 to establish distinct cell lines using different approaches.

The JE-KRT224 cell line was established via single-colony picking. Viable cells, initially collected from primary cultures using the scratch method, were counted and seeded into six multicell culture dishes (Cat. No. 76013-898, VWR International, Radnor, PA, USA), at a density of 5 cells/cm^2^ (405 cells/dish). The growing cells were observed periodically. Six colonies that were confirmed to be derived from a single cell and to have good growth were manually picked and isolated. Then, one colony showing good growth was sequentially expanded into 12-well plates, 6-well plates, T25 flasks, T75 flasks, and T300 flasks. JE-KRT224 cells were successfully cultured for more than 25 PDL following their establishment.

The JE-EK9 cell line was generated using the limiting dilution method. Cells collected using the scratch method were initially cultured in a 48-well plate. Cells from a well exhibiting robust growth (selected from approximately 17 initially seeded wells) were harvested by trypsinization, counted, and then subjected to single-cell cloning by seeding at a theoretical density of 0.4 cells/well into five 96-well plates. Initially, 12 clones were expanded from the resulting colonies into six-well plates, and then in T25, T75, and T300 flasks. One of these clones, JE-EK9, was successfully established and cultured for more than 25 PDL.

Finally, the JE-F1140 cell line was established from a long-term stationary culture. Primary cell cultures, initially derived from tissue explants, were maintained in growth medium in T75 flasks for over 19 months. During this period, distinct cell clumps formed. These clumps were transferred to a new T25 flask. The cells that migrated from these clumps (an initial harvest of 381 cells) were collected, cultured in separate T25 flasks, and expanded to T75 and T300 flasks. This process led to the establishment of the JE-F1140 cell line, which has since been cultured for more than 20 PDL.

All established cell lines were subsequently subcultured for further experiments or cryopreserved as described above.

### Population doubling time and cell diameter measurements

The automated cell counter was used to count total cell numbers (live and dead) and cell diameter (Countess™ 3 FL, Thermo Fisher Scientific). The PDL and population doubling time (PDT) were calculated using the following equations:

PDL = (logN−logN0)/log2; PDT = log2 × ∆t/(logN−logN0), where N = total cell number harvested; N0 = Initial number of seeded cells.

### Zeta-potential measurements

The cells (JE-KRT224, 119 PDL; JE-EK9, 98 PDL; JE-F1140, 109 PDL) were treated with 0.25% trypsin-EDTA to prepare a single-cell suspension and washed twice with PBS (-). Then, the cells were dispersed in PBS (-) and diluted 100-fold with distilled water. The ζ-potential measurements were performed using Omega cuvettes (Anton Paar, Graz, Austria) and a LiteSizer 500 (Anton Paar). Overall, three measurements with at least 100 runs each were performed at 25 °C with an adjusted voltage of 200 V, and the ζ-potential was calculated using the Helmholtz–Smoluchowski equation^49^.

### Cell proliferation under various FBS and fatty acid concentrations

To determine the effect of FBS concentration on cell growth, cells (JE-KRT224; 64 PDL) were seeded in 96-well plates at 1 × 10^4^ cells/well, cultured in growth medium for 2 days, and then cultured in a medium containing varying concentrations of FBS (0, 1, 2, 5, and 10%) for 5 days at 25 °C. Cell proliferation was measured using a Cell Counting Kit-8 (DOJINDO, Kumamoto, Japan). Absorbance intensity was measured using a microplate reader (Synergy HT, BioTek, Winooski, USA).

To determine the effect of fatty acid concentration on cell growth, cells (JE-KRT224, 81 PDL) were seeded in 12-well plates at 1 × 10^5^ cells/well, cultured in growth medium for 2 days, and then cultured in growth medium or growth medium supplemented with oleic acid (6.56, 16.4, 32.8, 65.6, and 164 μM) for 7 days at 25 °C. The cells were washed twice with PBS (-), treated with trypsin solution to dissociate them into single cells, which were counted using a Countess™ 3 FL cell counter. Oleic acid was added by diluting a solution of oleic acid dissolved in 1% solution of Ɣ-cyclodextrin in PBS (oleic acid–cyclodextrin complex; OACD).

To confirm the effect of fatty acid addition on cell proliferation, cells (JE-KRT224, 119 PDL; JE-EK9, 98 PDL; JE-F1140, 109 PDL) were seeded in 12-well plates at 5 × 10^4^ cells/well, cultured in growth medium for 2 days, and then cultured in growth medium or growth medium supplemented with 32.8 μM OACD for 4 weeks or 6 weeks at 25 °C. The cells were treated with 0.25% trypsin-EDTA to prepare a single-cell suspension and then counted (Countess™ 3 FL).

### Lipid accumulation, uptake, and fatty acids composition

To compare lipid accumulation in cells cultured with or without fatty acids, cells (JE-KRT224, 119 PDL; JE-EK9, 98 PDL; JE-F1140, 109 PDL) were seeded in 96-well black plates at a density of 2 × 10^4^ cells/well, cultured in growth medium for 2 days, and then cultured in growth medium or growth medium supplemented with 32.8 μM OACD for 1 or 3 weeks. Cells were washed twice with PBS (-), and lipid accumulation was measured using a lipid droplet assay kit (Cat. LD06, DOJINDO). Fluorescence intensity was measured using a fluorescence microplate reader (excitation 590 nm, emission 645 nm, Synergy HT).

To compare fatty acid uptake in cells cultured with or without fatty acids, cells (JE-KRT224, 119 PDL; JE-EK9, 98 PDL; JE-F1140, 109 PDL) were seeded in 96-well plates at 2 × 10^4^ cells/well, cultured in growth medium for 2 days, and then cultured in growth medium or growth medium supplemented with 32.8 μM OACD for 3 days. Fatty acid uptake was measured using a fatty acid uptake assay kit (Cat. UP07, DOJINDO). For the OACD group, 32.8 μM OACD was added to the assay medium. Cells were washed twice with PBS (-) and stained for 3 h at 25 °C. Fluorescence intensity was measured using a fluorescence microplate reader (Synergy HT) at an excitation wavelength of 480 nm and emission wavelength of 525 nm. The uptake of the fluorescent probe was observed using a Nikon AX microscope (Nikon Corp., Tokyo, Japan).

To measure fatty acid composition, cells (JE-KRT224, 108 PDL; JE-EK9, 88 PDL; JE-F1140, 98 PDL) were seeded in a T75 flask at 1 × 10^6^ cells, cultured in a growth medium for 2 days, and then cultured in a growth medium supplemented with 32.8 μM OACD for 12 days. Cells were collected using a cell scalper, washed twice with PBS (-), and stored at −80 °C. Fatty acid extraction and analysis were conducted at DOJIGLOCAL (Kumamoto, Japan).

### Immunofluorescence staining

Cells (JE-KRT224, 31 PDL; JE-EK9, 27 PDL; JE-F1140, 26 PDL) were cultured in a collagen-coated eight-well glass chamber slide (Matsunami, Tokyo, Japan) in growth medium until they reached 70%–80% confluence. These cells were fixed with ice-cold methanol for 15 min, permeabilized, and blocked in PBS (-) containing 0.3% Triton X-100 with 5% BSA overnight at 4 °C. Immunostaining was performed using the following primary antibodies: anti-ITGB1; CD29 (1:1000, Cat. PA5-29606, Thermo fisher scientific), anti- CD73 (1:500; Cat. 12231-1-AP; Proteintech), anti-Endoglin/CD105 (1:500; Cat. 16643-1-AP; Proteintech), anti-PAX7 (1: 200, Concentrate; DSHB), anti-MyoD (1:200, Cat. MA5-12902, Thermo Fisher Scientific), and anti-peroxisome proliferator-activated receptor gamma (PPARG; 1:200, Cat. 16643-1-AP, Proteintech) and anti-FABP4 (1:200; Cat. 12802-1-AP, Proteintech) overnight at 4 °C. The cells were then washed and incubated with a secondary antibody: rabbit IgG (H+L) highly cross-adsorbed secondary (1:2000, Cat. A-11034, Thermo Fisher Scientific for CD29, CD73, and CD105) or goat anti-mouse IgG1 cross-adsorbed secondary antibody (1:2000, Cat. A-21121, Thermo Fisher Scientific for PAX7 and MYOD) for 1 h at 25 °C in the dark. Vimentin was stained using a CoraLite555-conjugated vimentin recombinant antibody (1:500, Cat. CL555-80232; Proteintech) for 1 h at room temperature in the dark. The nuclei were stained with DAPI (1:1000, Cat. 62248, Thermo Fisher Scientific). The stained cells were visualized using a laser scanning confocal microscope (Nikon AX; Nikon Corp., Tokyo, Japan).

### Changes in *PPARG* gene expression

Cells (JE-KRT224, 31 PDL; JE-EK9, 27 PDL; JE-F1140, 26 PDL or JE-KRT224, 102 PDL; JE-EK9, 81 PDL; JE-F1140, 91 PDL for adipogenic differentiation) were seeded at 1 × 10^5^ cells/well in 12-well plates and cultured in growth medium for 2 days, and then cultured in growth medium, growth medium supplemented with 32.8 μM OACD, or adipogenic medium for 3 days. Cells were washed twice with PBS (-), and total RNA was extracted using RNeasy® Plus Mini Kit (Qiagen) and reverse transcribed to cDNA using ReverTra Ace® qPCR RT Master Mix with gDNA Remover (TOYOBO, Osaka, Japan). The expression of genes involved in adipogenic differentiation (*PPARG*) and glyceraldehyde-3-phosphate dehydrogenase (*GAPDH*) was examined using Brilliant III Ultra-Fast SYBR Green QPCR Master Mix (Agilent Technologies, Santa Clara, CA, USA) in a QuantStudio 3 real-time PCR detection system (Thermo Fisher Scientific)^50^. Relative gene expression was calculated using a standard curve generated from serially diluted standard samples, and *PPARG* transcript levels were normalized to those of *GAPDH*. Primers for *PPARG* were designed using Primer-BLAST based on the predicted sequences of Japanese eels in the NCBI database. The forward primer used was 5′-GAGATCGGGGTGCACGTCTT-3′, and the reverse primer was 5′- AGCAGCGTCACCTGGTCATT-3′.

### Differentiation potential to adipocytes, myotubes, cartilage, and osteoblasts

The differentiation potential of the cells was also examined. Cells (JE-KRT224, JE-EK9, and JE-F1140) were seeded in 24-well plates at 5 × 10^5^ cells/well and cultured in growth medium for 2 days, followed by induction.

Adipocyte: Cells (JE-KRT224, 31 PDL; JE-EK9, 27 PDL; JE-F1140, 26 PDL) were cultured in a growth medium or growth medium with 5 μg/mL insulin (Cat. 099-06473, FUJIFILM Wako Pure Chemical Corporation), 0.5 mM 3-isobutyl-1-methylxanthine (IBMX, Cat. 099-03411, FUJIFILM Wako Pure Chemical Corporation), 2.5 μM dexamethasone (Cat. 041-18861, FUJIFILM Wako Pure Chemical Corporation), and 32.8 μM OACD. Cells were fixed and stained with Oil Red O (154-02072, FUJIFILM Wako Pure Chemical Corporation) to visualize neutral lipids after 10 days of lipid accumulation.

To observe adipocyte differentiation, cells (JE-KRT224; 77 PDL) were seeded in a glass-bottom dish at 2 × 10^4^ cells/well, cultured in growth medium for 2 days, and then cultured in adipogenic medium. Time-lapse images of autofluorescence signals were recorded using an inverted Ti2 Eclipse microscope every 30 min for 60 h (Excitation, 488 nm; Emission, 500–550 nm, and TD, Nikon Corp.).

To determine lipid accumulation in adipocytes, cells (JE-KRT224, 52 PDL; JE-EK9, 34 PDL, and JE-F1140, 45 PDL) were seeded in 96-well black plates at 2 × 10^4^ cells/well, cultured in growth medium for 2 days, and then cultured in growth medium or adipogenic medium for 7 days. Cells were washed twice with PBS (-) and lipid accumulation was measured using a Lipid Droplet Assay Kit (Cat. LD05, DOJINDO). Fluorescence intensity was measured using a fluorescence microplate reader (excitation 360 nm, emission 460 nm, Synergy HT).

Myotube: Cells (JE-KRT224, 31 PDL; JE-EK9, 27 PDL; JE-F1140, 26 PDL) were grown in L-15 medium containing 2% horse serum (Cat. 16050130, Thermo Fisher) for 10 days and examined microscopically for myotubular differentiation^25^.

Cartilage: Cells (JE-KRT224, 48 PDL; JE-EK9, 31 PDL; JE-F1140, 43 PDL) were cultured in a growth medium containing 10 ng/mL transforming growth factor beta 1 (TGF-β1, Oriental Yeast Co., Ltd., Tokyo, Japan), 6.25 μg/mL insulin supplemented with or without 100 nM dexamethasone for 6 weeks. The cells were fixed with 4% PFA and cartilage formation was determined by Alcian Blue (pH 2.5) staining^26^.

Osteoblast: Cells (JE-KRT224, 48 PDL; JE-EK9, 31 PDL; JE-F1140, 43 PDL) were cultured in a growth medium supplemented with 100 nM dexamethasone, 1 mM β-glycerophosphate (Cat. 048-34332, FUJIFILM Wako Pure Chemical Corporation), and 0.2 mM ascorbic acid (Cat. 013-12061, FUJIFILM Wako Pure Chemical Corporation) for 6 weeks. Following this, the cells were fixed with 4% PFA and mineralization was determined by alizarin red staining^26^.

The medium was changed every 3 or 4 days for adipocyte and myotube differentiation, and every 5–7 days for osteoblast and cartilage differentiation.

### Statistical analyses

Data are presented as mean ± SD. Student’s t-test was used to determine the significance of the differences between two groups, as indicated in the figure legends. For the comparison of more than two groups, one-way Analysis of Variance (ANOVA) was performed, as indicated in the figure legends, with multiple comparisons performed using Tukey’s honest significant difference (HSD) post-hoc test or Dunnett’s test. Statistical significance was set at p < 0.05.

## Supplementary information

Supplementary information

## Data Availability

All relevant data supporting this study are included within the manuscript and supplementary data. The spontaneously immortalized cell lines used in this study are available to non-profit researchers through the National Institute of Technology and Evaluation (NITE) in Japan. (JE-KRT224; NITE BP-04244, JE-EK9; NITE BP-04246, and JE-F1140; NITE BP-04247).
