# Supplementary material for: Establishment of spontaneously immortalized Japanese eel muscle-derived preadipocyte cell lines for cultured seafood production
Source: NPJ Sci Food. 2025 Nov 20;9:240. doi: 10.1038/s41538-025-00557-x (PMC12635220; doi:10.1038/s41538-025-00557-x)
Supplement: Supplementary file 1 — Supplementary information [file 41538_2025_557_MOESM1_ESM.docx]

**Supplementary information**

**
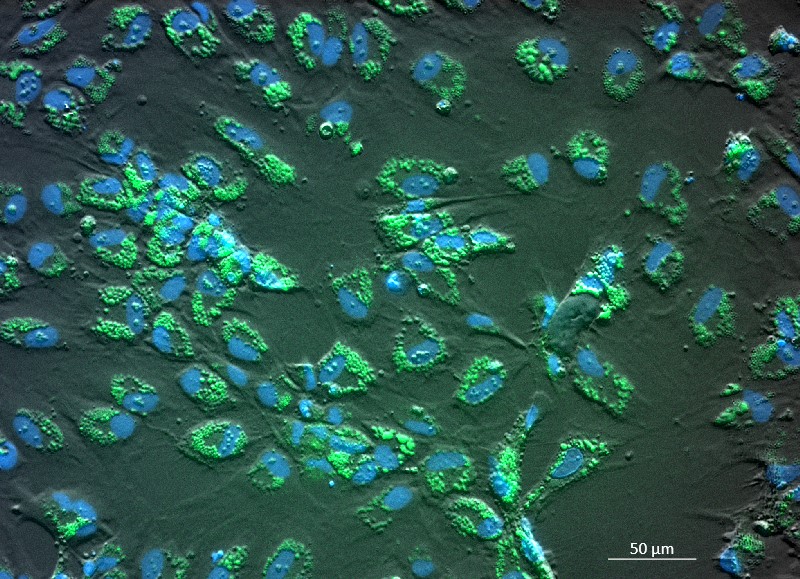
**

**Supplementary Figure. 1.** Primary cultured cells of the Japanese eel were seeded in a glass-bottom dish and cultured in a growth medium for 13 days. Fluorescently stained primary cultured cells were visualized using Celldiscoverer 7 (ZEISS), with nuclei and lipids stained with Hoechst 33342 (blue) and Lipi-Green (Excitation, 488 nm / Emission, 500–550 nm), respectively. Scale bars 50 μm.

**Supplementary Figure. 2.** Potential for differentiation to myotubes, cartilage, and osteoblasts. **A** Potential for differentiation to myotubes. None of the cell lines differentiated into myotubes, whereas primary cultured cells showed myotube formation. Scale bars, 200 μm. **B** Potential for differentiation to cartilage. Based on Alcian Blue (pH 2.5) staining, no clear differences were observed compared with GM, regardless of the presence or absence of dexamethasone (DEX). Scale bars 500 μm. **C** Differentiation potential for osteoblasts. Osteoblasts stained red with alizarin red staining, but no red cells or sections were observed. Scale bars, 200 μm.

Supplementary Movie


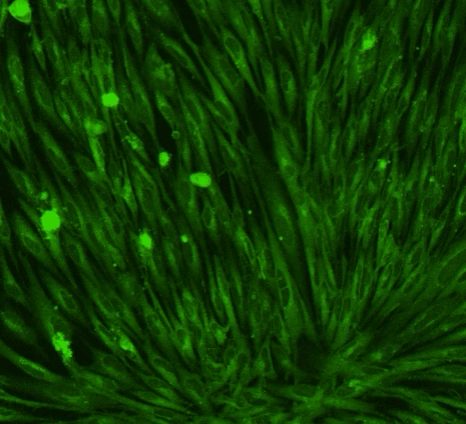


**Supplementary Movie 1.** Cells (JE-KRT224) were seeded in a glass-bottom dish, cultured in growth medium for 2 days, and then cultured in adipogenic medium (growth medium supplemented with 5 μg/mL insulin, 0.5 mM 3-isobutyl-1-methylxanthine, 2.5 μM dexamethasone, and 32.8 μM oleic acid–cyclodextrin complex). Time-lapse images of autofluorescence signals were recorded using an inverted Ti2 Eclipse microscope (Nikon Corp.) every 30 min for 60 h (Ex: 488 nm, Em: 500–550 nm).
